# Comparative Analysis of Small Nerve Fiber Density in Fibromyalgia Syndrome and Small Fiber Neuropathy

**DOI:** 10.3390/biomedicines13092109

**Published:** 2025-08-29

**Authors:** Pietro Falco, Eleonora Galosi, Caterina Maria Leone, Gianfranco De Stefano, Giuseppe Di Pietro, Giulia Di Stefano, Nicoletta Esposito, Enrico Evangelisti, Daniel Litewczuk, Cristina Mollica, Lars Arendt-Nielsen, Andrea Truini

**Affiliations:** 1 Department of Human Neuroscience, Sapienza University, 00185 Rome, Italy; 2 Department of Statistical Sciences, Sapienza University, 00185 Rome, Italy; 3 Centre for Neuroplasticity and Pain, Department of Health Science and Technology, School of Medicine, Aalborg University, 9000 Aalborg, Denmark; 4 Department of Gastroenterology & Hepatology, Mech-Sense, Clinical Institute, Aalborg University Hospital, 9000 Aalborg, Denmark; 5 Steno Diabetes Center North Denmark, Clinical Institute, Aalborg University Hospital, 9000 Aalborg, Denmark

**Keywords:** fibromyalgia syndrome, small fiber neuropathy, skin biopsy, intraepidermal nerve fiber density, small fiber pathology

## Abstract

**Background/Objectives:** Fibromyalgia syndrome is commonly associated with reduced intraepidermal nerve fiber density (IENFD), as assessed by skin biopsy, a finding referred to as small fiber pathology (SFP-FMG). The clinical significance of this abnormality, and how it relates to symptoms in fibromyalgia, remains uncertain. Reduced IENFD also represents the defining feature of small fiber neuropathy (SFN). While previous observations suggest that IENFD reduction is generally less severe in SFP-FMG than in SFN, no study has directly confirmed this finding in a large cohort. This retrospective study aimed to compare the severity of IENFD reduction in patients with SFP-FMG and those with SFN. **Methods:** To account for age and sex differences, we used the age-and sex-adjusted axonal loss density (ALD), defined as the percentage reduction from normative IENFD values. We retrospectively analyzed skin biopsy data from 73 patients with SFP-FMG and 134 patients diagnosed with SFN. **Results:** We found that the reduction in IENFD was significantly milder in patients with SFP-FMG than in those with SFN both at distal and proximal sites. Receiver operating characteristic analysis indicated that an ALD threshold of 37.6% provided good specificity for distinguishing SFN from SFP-FMG. **Conclusions:** These findings indicate that small fiber damage in fibromyalgia syndrome is quantitatively mild compared to patients with SFN. This may explain the absence of detectable sensory deficits on clinical examination and suggests a limited contribution of peripheral nerve damage to the pathophysiology of fibromyalgia syndrome.

## 1. Introduction

Fibromyalgia syndrome is a clinical condition characterized by widespread chronic pain, accompanied by symptoms such as depression, sleep disturbances, and brain fog [1]. Biopsy studies on skin samples have shown that approximately 30–50% of fibromyalgia patients exhibit a reduction in intraepidermal small fibers [2]. However, despite this finding, fibromyalgia patients do not display the typical clinical and functional alterations observed in individuals with small fiber neuropathy (SFN). For this reason, the condition has been referred to as small fiber pathology (SFP) [3]. The clinical significance of SFP in patients with fibromyalgia (SFP-FMG) and its role in the pathophysiology of fibromyalgia syndrome remain uncertain.

Although fibromyalgia syndrome and painful small fiber neuropathy (SFN) have distinct clinical profiles, some SFN patients can also meet diagnostic criteria for fibromyalgia syndrome [4,5]. In this context, skin biopsy has limited discriminative value, as both conditions may present with reduced intraepidermal nerve fiber density (IENFD).

Previous studies have already demonstrated that the IENFD reduction distribution differs between the two conditions. SFN patients exhibit more frequently a length-dependent reduction in small fibers [5], whereas SFP-FMG patients more frequently show a non-length-dependent pattern [6]. Moreover, a previous study from our group reported, as a secondary finding, a milder reduction in IENFD in SFP-FMG compared to SFN [7]. Although observed in a limited population, this finding suggested that the two groups may also differ quantitatively, warranting confirmation in a larger cohort.

This study aims to retrospectively compare IENFD between patients with SFP-FMG and those with SFN in a large cohort. Demonstrating that IENFD reduction is less severe in SFP-FMG compared to SFN could enhance our understanding of how small fiber damage contributes to clinical expression in patients with fibromyalgia syndrome. Additionally, defining indicative ranges that help discriminate between the two conditions could potentially assist in detecting SFN in patients with overlapping fibromyalgia syndrome.

## 2. Materials and Methods

### 2.1. Study Cohort and Design

We retrospectively examined skin biopsy data collected at the Peripheral Neuropathy and Neuropathic Pain Unit at the Department of Human Neuroscience of Sapienza University, Rome, from January 2019 to December 2024, and selected patients diagnosed for fibromyalgia syndrome or SFN, having distal IENFD reduction.

In a single clinical session, these patients underwent an extensive clinical examination and medical history recording, nerve conduction study (NCS), cold and warm detection thresholds measured by quantitative sensory testing (QST), and skin biopsy at distal and proximal site.

Exclusion criteria were age under 18 years, major psychiatric comorbidities, central nervous system diseases, cognitive impairment, and communication barrier, as assessed with clinical history and examination.

For the purpose of this retrospective study, we included in the SFP-FMG group patients with fibromyalgia syndrome diagnosed according to the widely accepted American College of Rheumatology criteria [4], and with reduced IENFD at the distal site (calf) of the lower limb, based on a previously published multicenter, multinational normative dataset using immunofluorescent staining, adjusted for age and gender [8].

We included in the SFN population patients diagnosed with SFN according to Besta criteria [9]. Among them, we selected patients with reduced IENFD at the distal site (calf) of the lower limb, based on worldwide normative ranges adjusted for age and gender and no NCS or clinical signs of large-fiber damage. For the purposes of the study, we did not include patients who met the diagnostic criteria for both conditions simultaneously.

The main outcome measure was IENFD at the distal site in patients with SFP-FMG and in patients with SFN. To account for the different IENFD reduction distribution in the two conditions, we also conducted a subgroup analysis comparing proximal sites in patients from both groups who exhibited a histologically determined non-length-dependent IENFD reduction (leg/thigh ratio > 0.48) [10].

To account for differences in IENFD across various age and sex groups, we used the axonal loss degree (ALD) as a normalized variable for assessing changes in IENFD. ALD was calculated as the percentage of intraepidermal nerve fiber loss relative to the age-and sex-corrected lower limit of IENFD, according to the following formula: ALD = −(Normal IENFD cutoff − Patient IENFD) × 100/Normal IENFD cutoff. Normal IENFD cutoff refers to age-and sex-specific normative IENFD values obtained through the immunofluorescence technique. Accordingly, positive ALD values indicate IENFD below the normal range [8,10,11,12].

### 2.2. Clinical Examination and Diagnostic Tests

All subjects included in the study underwent detailed medical history recording and neurological examination. Touch and pinprick perception were assessed using a piece of cotton wool and a wooden cocktail-stick, as recommended [13]. For the cold detection, a metal rod at 22 °C room temperature was applied for 3 s. For the warm detection, a glass vial was applied for 3 s to the skin after having been warmed within the examiner’s hands at body temperature (36–37 °C). Vibration perception was measured with a 128 Hz graduated Rydel–Seiffer tuning fork. Patients were instructed to identify any pain caused by normally non-painful touch (allodynia) or an exaggerated pain response to pinprick stimuli (hyperalgesia). Deep tendon reflexes were assessed and graded as normal, decreased, or absent. We used the Medical Research Council (MRC) score to grade muscle strength [13].

Patients also underwent sensory and motor NCS using surface recording electrodes with standard placement, with recording methods adhering to the recommendations of the International Federation of Clinical Neurophysiology [14]. We recorded sensory nerve action potential and conduction velocity from sural, ulnar, and superficial radial nerves, and compound motor action potential amplitude and conduction velocity of peroneal, tibial, and ulnar nerves. Skin temperature was maintained between 34 °C and 36 °C. Data were compared with age-adjusted normative ranges [15].

We also tested cold and warm detection thresholds using the Medoc™ device (Medoc™ Thermal Sensory Analyzer, TSA-2001, Ramat Yishai, Israel), following the instructions of the QST protocol of the German Research Network on Neuropathic Pain (DFNS) [16]. Cold and warm detection thresholds were quantified with the method of limits, with a 30 × 30 mm large probe delivering ramped thermal stimuli at 1 °C/s from a baseline of 32 °C. Absolute reference data (specific for body region, age, and gender) were used to normalize test results of individual patients by calculating the z-transform: Z = (value patient − mean controls)/SD controls [16]. The dorsum of the foot was used as test site, being the most painful area in many patients with polyneuropathies [17].

### 2.3. Skin Biopsy

Patients underwent skin biopsy at the distal leg, 10 cm above the lateral malleolus, and from the lateral upper thigh, 20 cm under the antero-superior iliac spine, using a 3 mm disposable circular punch after local lidocaine anesthesia. Biopsies were performed under sterile conditions and with no suture required.

Using indirect immunofluorescence, intraepidermal innervation was assessed with the pan-neuronal marker PGP9.5. Biopsies were fixed for 24 h at 4 °C in Zamboni’s fixative, then were cryoprotected overnight with a solution containing 30% (*v*/*v*) ethylene glycol and 30% (*w*/*v*) sucrose in phosphate-buffered saline with 1% polyvinylpyrrolidone (PVP). A cut was performed at −23 °C with a cryostat (MEV, SLEE medical GmbH, Nieder- Olm, Germany) to obtain 50 µm-thick sections. Three non-consecutive free-floating sections were randomly selected for immunostaining from each sample and blocked with 5% normal donkey serum for 1 h. Sections were then incubated overnight with a rabbit anti-human PGP9.5 monoclonal antibody (Abcam, Cambridge, UK, 1:500 diluted) and a mouse anti-human collagen IV monoclonal antibody (Millipore, Darmstadt, Germany, 1:1600). The following day, sections were incubated with anti-rabbit-Cy3 (Jakson ImmunoResearch, Cambridgeshire, UK, 1:800) and anti-mouse-488 (Jakson ImmunoResearch, Cambridgeshire, UK, 1:400) secondary antibodies overnight.

We calculated IENFD according to European Federation of Neurological Societies and Peripheral Nerve Society guidelines [18]. Epidermal linear length was measured through Image-J to obtain a linear density (number of fibers/mm). Normative values from an internationally recognized dataset were applied [8]. All skin biopsies were collected and processed in the laboratory of the Peripheral Neuropathy and Neuropathic Pain Unit at the Department of Human Neuroscience, Sapienza University. The IENFD analysis of the skin biopsy samples was performed by two blinded operators (PF, EG).

### 2.4. Statistical Analysis

In the preliminary univariate analysis, we first described the sample’s clinical characteristics by reporting means ± SD and percentage frequencies for numerical and categorical variables, respectively. The assumption of normality was assessed using the D’Agostino and Pearson omnibus test. We used *t* test, or the Mann–Whitney test if the normality assumption was rejected, to evaluate differences in quantitative demographic, QST, and skin biopsy data between patients with SFN and those with SFP-FMG. The association with categorical variables was evaluated with the Chi-square or Fisher’s exact test, as appropriate.

Additionally, we investigated the discriminative ability of ALD at the distal site for distinguishing between the two groups to identify a threshold above which the probability of having small fiber neuropathy is higher and therefore warrants further investigation and follow-up. To do so, we estimated the area under the receiver operating characteristic (ROC) curve. In the ROC curve analysis, we compared the ALD at the distal site between patients with SFN and patients with SFP-FMG. The optimal cut-off of the ALD was determined by maximizing the Youden index (sensitivity + specificity − 1) derived from the ROC curve analysis. The estimated sensitivity, specificity, positive predictive value (PPV), and negative predictive value (NPV) of the optimal ALD threshold were reported with the related 95% confidence intervals (CIs) to assess the discriminating ability of the resulting ALD classifier.

All tests were two-sided, with a *p*-value ≤ 0.05 considered statistically significant. Statistical methods were implemented using R statistical software version 4.4.2. The graphs were created using GraphPad Prism 10.5.

## 3. Results

We included data from 196 patients, 62 with SFP-FMG and 134 with SFN (Table 1).

Among the 62 patients with SFP-FMG, 44 (71%) had a non–length-dependent distribution of IENFD reduction, with a leg/thigh ratio above 0.48. All included patients showed no clinically evident thermal-pain hypoesthesia on neurological examination and normal thermal thresholds on QST.

We included 134 patients with SFN showing a combination of IENFD reduction at skin biopsy from the distal calf associated with distally distributed clinical negative sensory signs and/or abnormal cold and/or warm detection threshold as assessed by QST. Among these 134 patients, 63 (46%) had a non–length-dependent distribution of IENFD reduction, with a leg/thigh ratio above 0.48.

In the 134 patients, SFN was associated with multiple etiologies (25 diabetes, 30 autoimmune diseases, 12 infectious diseases, 38 genetically determined, 29 idiopathic).

Patients with FMG-SFP were younger than those with SFN (*p* = 0.0002).

Cold and warm detection thresholds (CDT and WDT), reported as normalized z-scores in Table 1, were lower in patients with SFN (*p* < 0.001; *p* = 0.001).

### Skin Biopsy Findings

**Figure 1:**
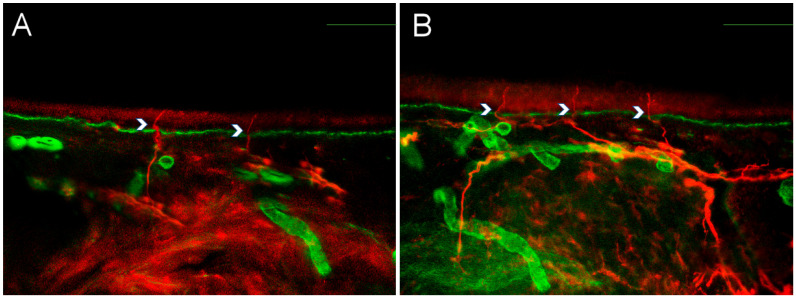
Intraepidermal nerve fiber density in small fiber neuropathy and small fiber pathology. Representative pictures from a patient with small fiber neuropathy (**A**) and a patient with fibromyalgia-related small fiber pathology (**B**), showing nerve fiber counting examples for intraepidermal nerve fiber density (IENFD). Red staining represents PGP9.5, marking nerve fibers, while green staining corresponds to collagen IV. Calibration bars: 100 μm.

**Figure 2:**
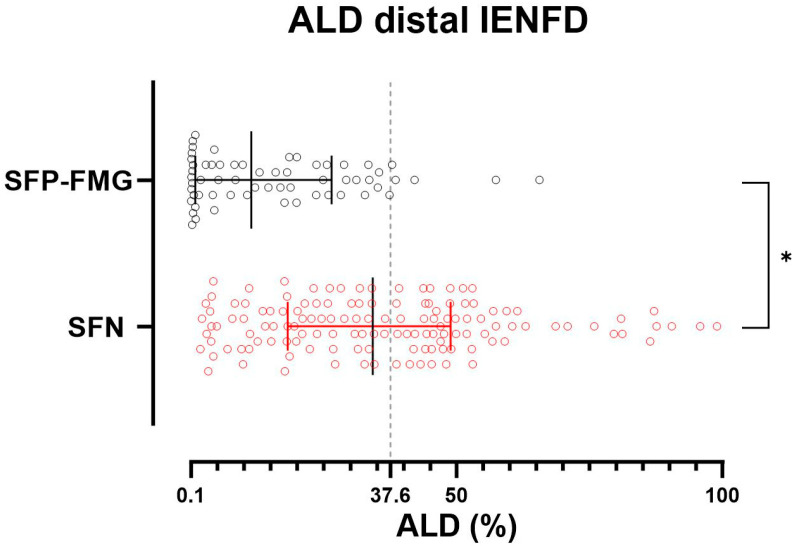
Reduction in IENFD at the distal site in patients with SFP-FMG and SFN. Each dot represents the axonal loss degree (ALD) value in patients with small fiber neuropathy (SFN) in red and patients with fibromyalgia-related small fiber pathology (SFP-FMG) in black. ALD is calculated as the percentage loss of PGP 9.5-immunostained fibers relative to the age- and sex-corrected lower limit of intraepidermal nerve fiber density, which is set at 0. A significant proportion of patients with fibromyalgia-related small fiber pathology have an ALD close to 0. The grey dotted line represents the ALD threshold with higher discriminative ability. A Mann–Whitney test was used to compare ALD between SFP-FMG and SFN groups (* = *p* < 0.0001). Median and interquartile range are reported.

The axonal loss degree (ALD) at the distal site was significantly higher in patients with SFN than in those with SFP-FMG (SFN = 36.13 ± 22.32; SFP-FMG = 15.53 ± 15.61) (Figure 1 and Figure 2) (*p* < 0.0001) (Tables S1 and S2). The subgroup analysis by etiology shows that the difference in distal ALD between the different SFN subgroups and patients with SFP-FMG is maintained, except for the subgroup with SFN associated with infectious diseases (Table S3).

**Figure 3:**
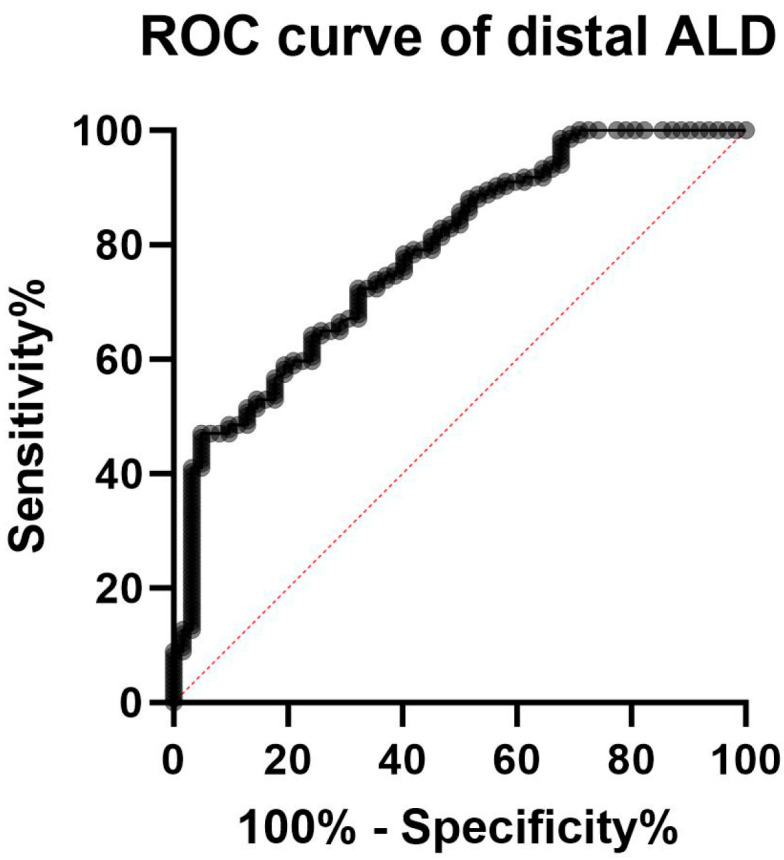
ROC curve for the accuracy of intraepidermal nerve fiber density reduction for distinguishing fibromyalgia-related small fiber pathology and small fiber neuropathy. The AUC of the ROC curve of 0.81 (95%CI 0.74 to 0.87, *p* < 0.0001) indicated that the difference in intraepidermal nerve fiber density reduction between patients with small fiber neuropathy and those with fibromyalgia-related small fiber pathology has a good discriminatory ability (with a maximized Youden index corresponding to an ALD of 37.6%).

The ROC curve analysis showed an AUC of 0.81 (95%CI 0.74 to 0.87, *p* < 0.0001) (Figure 3). According to the maximized Youden index, at the distal site an ALD value of 37.6% was the optimal threshold to discriminate between the two groups, with a specificity of 91% (95%CI 0.818 to 0.958) and a sensibility of 50% (95%CI 0.412 to 0.587), with 0.91 positive (95%CI 0.82 to 0.96) and 0.46 negative predictive value (95%CI 0.38 to 0.55).

The subgroup analysis on patients with non–length-dependent distribution disclosed results comparable to the principal analysis. The axonal loss degree (ALD) at the proximal site was significantly higher in patients with SFN than in those with SFP-FMG (SFN = 33.63 ± 22.41; SFP-FMG = 12.32 ± 23.03) (Figure S1) (*p* < 0.0001).

## 4. Discussion

In this retrospective study, we found that the IENFD reduction was less severe in patients with SFP-FMG compared to those with SFN. The milder IENFD reduction observed in patients with fibromyalgia syndrome may help explain the absence of clear sensory deficits on neurological examination and may potentially aid in the recognition of SFN in patients with overlapping fibromyalgia syndrome.

To account for the influence of age and sex on IENFD, we expressed fiber loss using the ALD. The ALD value was calculated as the percentage loss of PGP 9.5-immunostained fibers relative to the age-and sex-corrected lower limit of IENFD [8,10,11,12]. This metric allowed for a standardized comparison across patients. We believe that the use of ALD as a normalized index offers a robust method for adjusting IENFD values by age and sex, and may serve as a reliable tool for future comparative studies.

In the analysis conducted to differentiate ALD values between patients with FMG-SFP and SFN, we included only patients who had abnormal IENFD. This approach was deemed more appropriate, as it allows for a comparison between two patient populations with distinct clinical conditions but a shared skin biopsy abnormality.

Our results, derived from a large patient cohort, confirm previous evidence that small fiber density is quantitatively less reduced in SFP-FMG than in SFN [7], according to the ALD value calculation. The mild IENFD reduction in patients with fibromyalgia likely explains the absence of clear sensory deficits and the subtle abnormalities in small fiber function tests in this group [6,19]. Previous studies have shown that the degree of IENFD reduction in polyneuropathies correlates with both objective and psychophysical measures of nerve function [20], supporting the idea that milder morphological changes may result in minimal or clinically undetectable neurological signs and symptoms.

The relationship between SFP, sensory disturbances, and pain in fibromyalgia syndrome remains controversial. Previous studies have shown that the abnormal IENFD in fibromyalgia does not correlate with clinically evident sensory disturbances or QST variables [19,21]. Thus, the role of SFP in fibromyalgia syndrome remains unclear, and the contribution of peripheral small fiber damage to the widespread pain experienced by these patients might be negligible. The difference in the severity of IENFD reduction, as assessed using ALD values, consistently highlights that fibromyalgia syndrome and SFN, despite potentially sharing similar symptoms (e.g., pain and autonomic dysfunction) are distinct clinical entities with different underlying mechanisms and treatment strategies [6,19]. Our data, therefore, support the hypothesis that mechanisms of altered central modulation are more likely responsible for pain in fibromyalgia syndrome.

We found that ALD values differed significantly between patients with SFP-FMG and those with SFN. ROC curve analysis demonstrated good discriminatory ability, with the optimal Youden index corresponding to an ALD threshold of 37.6%. This threshold showed high specificity and positive predictive value for discriminating between the two groups, with an IENFD reduction exceeding 37.6% being more consistent with SFN. However, the relatively low sensitivity (47%) indicates that a considerable proportion of patients with SFN may have ALD values below this threshold, potentially leading to false-negative results. These findings are in line with current evidence suggesting that skin biopsy, when used in isolation, may have limited diagnostic accuracy. As such, the diagnosis of SFN should be based on a comprehensive clinical assessment incorporating neurological examination and multiple diagnostic tools, rather than relying solely on IENFD evaluation [22,23].

Nevertheless, our findings may help guide the interpretation of skin biopsy results in a clinical context. In some cases, SFN and fibromyalgia syndrome can coexist, and the overlap of symptoms such as sensory disturbances, autonomic dysfunction, and fatigue can complicate the identification of SFN in patients with fibromyalgia syndrome. Our finding that SFN is associated with a more significant IENFD reduction, corresponding to an ALD threshold greater than 37.6%, may warrant increased attention to the possibility of coexisting SFN in patients with fibromyalgia syndrome, which should prompt clinicians to pursue additional diagnostic steps, including laboratory screening and targeted follow-up.

Our inclusion criteria for patients with SFP-FMG required IENFD values below established normative thresholds specifically at the distal site, which allowed for a direct comparison with patients with SFN. However, several studies have reported reduced proximal IENFD in patients with fibromyalgia [3]. Therefore, we conducted a supplementary analysis that included data from proximal skin biopsy sites. This analysis was consistent with the main findings and showed that proximal IENFD values, assessed using ALD, were significantly lower in patients with SFP-FMG compared to those with SFN (Figure S1).

### Limitations

The retrospective design of the study represents a limitation, as retrospective data collection may introduce both selection and recall bias. These potential biases could distort the findings and affect the overall validity of the study. However, we implemented clearly defined inclusion and exclusion criteria and used robust data sources. Data collection was standardized, ensuring that all information was consistently and accurately recorded. Additionally, we relied on widely agreed diagnostic criteria, which currently represent the gold standard for diagnosing fibromyalgia syndrome and SFN [4,9].

We acknowledge that intraepidermal nerve fiber loss is a dynamic, rather than static, process influenced by disease duration and progression. Patients in both groups may be at varying stages of their condition, and those with longer-standing symptoms—particularly in the SFN group—are likely to exhibit greater cumulative nerve fiber loss. Consequently, a cross-sectional analysis of IENFD may offer only limited insights into disease mechanisms. However, we believe that data collected from a large sample of patients at different stages of disease progression can still yield meaningful comparative information, especially when interpreted alongside clinical context. Furthermore, a longitudinal study has shown that in patients with fibromyalgia syndrome, the small fiber loss does not substantially change over time [24].

Additionally, the SFN patient group includes patients with different etiologies, resulting in more heterogeneous data. However, subgroup analysis by etiology demonstrated that a statistically significant difference in ALD is maintained between the different etiological subgroups and the SFP-FMG patients, with the exception of the infection-related neuropathy subgroup, which is likely influenced by the limited sample size (12 patients) (Table S3).

Finally, in calculating ALD at the proximal site, sex-specific values were not used, as they are not currently available in the literature.

## 5. Conclusions

Our retrospective study found that patients with SFP-FMG had a milder reduction in IENFD compared to those with SFN, with an IENFD reduction corresponding to an ALD value greater than 37.6% being more consistent with a diagnosis of SFN. These findings may help enhance our understanding of how small fiber impairment contributes to clinical expression in fibromyalgia syndrome, potentially explaining the absence of sensory deficits on neurological examination, and may also assist in recognizing SFN in patients who have fibromyalgia syndrome.

## Figures and Tables

**Table 1:** Comparison of demographic, QST, and skin biopsy parameters between patients with SFP-FMG and SFN.

	Patients SFP-FMG (N = 62)	Patients with SFN (N = 134)	*p*
Age (years)	48.87 (11.34)	55.8 (12.64)	**0.0002**
WDT (z-score)	−0.5456 (1.41)	−1.312 (1.07)	**0.001**
CDT (z-score)	−0.9825 (0.8571)	−1.939 (1.195)	**<0.001**
Distal ALD (%)	15.53 (15.61)	36.13 (22.32)	**<0.0001**
Proximal ALD (%) (N = 44 SFP-FMG; N = 63 SFN)	12.32 (23.03)	33.63 (22.41)	**<0.0001**

Each value is expressed as mean (SD). QST: quantitative sensory testing; SFP-FMG: small fiber pathology in patients with fibromyalgia syndrome; SFN: small fiber neuropathy; WDT: warm detection threshold; CDT: cold detection threshold; ALD: axonal loss degree. Mann–Whitney test was used to compare age and ALD values. *t*-test was used to compare WDT and CDT values.

## Data Availability

The original contributions presented in this study are included in the article/supplementary material. Further inquiries can be directed to the corresponding author.

## Supplementary Materials

The following supporting information can be downloaded at: https://www.mdpi.com/article/10.3390/biomedicines13092109/s1, Table S1. Distal ALD calculation in patients with SFP-FMG. Table S2. Distal ALD calculation in patients with SFN. Table S3. Subgroup analysis of patients with SFN sorted by etiology. Figure S1. Subgroup analysis comparing intraepidermal nerve fiber density (IENFD) between patients with fibromyalgia-related small fiber pathology (SFP-FMG) and those with small fiber neuropathy (SFN) at the proximal site, limited to patients with a leg/thigh ratio above 0.48. Each dot represents the axonal loss degree (ALD) value in patients with SFN in red and patients with SFP-FMG in black. ALD is calculated as the percentage loss of PGP 9.5-immunostained fibres relative to the age- and sex-corrected lower limit of IENFD, which is set at 0. Mann-Whitney test was used to compare ALD between SFP-FMG and SFN group (* = *p* < 0.0001). Median and interquartile range are reported.
