# Supplementary material for: Comparative Analysis of Small Nerve Fiber Density in Fibromyalgia Syndrome and Small Fiber Neuropathy
Source: Biomedicines. 2025 Aug 29;13(9):2109. doi: 10.3390/biomedicines13092109 (PMC12467328; doi:10.3390/biomedicines13092109)
Supplement: Supplementary file 1 [file biomedicines-13-02109-s001.zip › Supplementary Table S1.pdf]

**Supplementary Table S1.** Distal ALD calculation in patients with SFP- FMG.

| | age | Sex (0=M, 1=F) | IENFD | NV cutoff | ALD |
|---|---|---|---|---|---|
| 1 | 36 | 0 | 6,33 | 10,3 | 38,54369 |
| 2 | 69 | 1 | 7,84 | 9,8 | 20 |
| 3 | 38 | 1 | 9,33 | 11,4 | 18,15789 |
| 4 | 57 | 1 | 8,482 | 10,3 | 17,65049 |
| 5 | 56 | 1 | 6,67 | 10,3 | 35,24272 |
| 6 | 60 | 1 | 7,36 | 9,8 | 24,89796 |
| 7 | 42 | 1 | 7,637 | 10,8 | 29,28704 |
| 8 | 32 | 1 | 11,38 | 11,4 | 0,175439 |
| 9 | 56 | 1 | 9,05 | 10,3 | 12,13592 |
| 10 | 40 | 1 | 10,75 | 10,8 | 0,462963 |
| 11 | 50 | 0 | 6,176 | 9,3 | 33,5914 |
| 12 | 51 | 1 | 9,45 | 10,3 | 8,252427 |
| 13 | 60 | 1 | 9,65 | 9,8 | 1,530612 |
| 14 | 35 | 1 | 11,35 | 11,4 | 0,438596 |
| 15 | 74 | 1 | 8,83 | 9,2 | 4,021739 |
| 16 | 45 | 1 | 9,4 | 10,8 | 12,96296 |
| 17 | 49 | 1 | 8,77 | 10,8 | 18,7963 |
| 18 | 60 | 1 | 9,75 | 9,8 | 0,510204 |
| 19 | 51 | 0 | 9,27 | 9,3 | 0,322581 |
| 20 | 48 | 1 | 8,798 | 10,8 | 18,53704 |
| 21 | 53 | 1 | 6,65 | 10,3 | 35,43689 |
| 22 | 52 | 1 | 7,87 | 10,3 | 23,59223 |
| 23 | 62 | 1 | 7,48 | 9,8 | 23,67347 |
| 24 | 52 | 1 | 9,84 | 10,3 | 4,466019 |
| 25 | 56 | 1 | 10,26 | 10,3 | 0,38835 |
| 26 | 36 | 1 | 11,3 | 11,4 | 0,877193 |
| 27 | 58 | 1 | 6,4 | 10,3 | 37,86408 |
| 28 | 36 | 1 | 8,11 | 11,4 | 28,85965 |
| 29 | 55 | 1 | 9,758 | 10,3 | 5,262136 |
| 30 | 23 | 1 | 11,56 | 11,9 | 2,857143 |
| 31 | 54 | 1 | 9,2 | 10,3 | 10,67961 |
| 32 | 39 | 0 | 5,96 | 10,3 | 42,13592 |
| 33 | 56 | 1 | 10,27 | 10,3 | 0,291262 |
| 34 | 39 | 1 | 7,59 | 11,4 | 33,42105 |
| 35 | 45 | 1 | 9,89 | 10,8 | 8,425926 |
| 36 | 44 | 0 | 8,65 | 10,3 | 16,01942 |
| 37 | 61 | 1 | 9,257 | 9,8 | 5,540816 |
| 38 | 54 | 1 | 8,25 | 10,3 | 19,90291 |
| 39 | 39 | 1 | 8,48 | 11,4 | 25,61404 |
| 40 | 59 | 1 | 10,2 | 10,3 | 0,970874 |
| 41 | 48 | 1 | 9,737 | 10,8 | 9,842593 |
| 42 | 29 | 1 | 8,81 | 11,9 | 25,96639 |
| 43 | 34 | 1 | 11,3 | 11,4 | 0,877193 |
| 44 | 24 | 1 | 10,89 | 10,9 | 0,091743 |
| 45 | 50 | 1 | 10,24 | 10,3 | 0,582524 |
| 46 | 63 | 1 | 7,03 | 9,8 | 28,26531 |
| 47 | 45 | 0 | 10,1 | 10,3 | 1,941748 |
| 48 | 65 | 1 | 9,36 | 9,8 | 4,489796 |
| 49 | 58 | 1 | 8,56 | 10,3 | 16,8932 |
| 50 | 46 | 1 | 6,76 | 10,8 | 37,40741 |
| 51 | 52 | 0 | 6,4 | 9,3 | 31,1828 |
| 52 | 29 | 0 | 10,88 | 10,9 | 0,183486 |
| 53 | 37 | 1 | 3,919 | 11,4 | 65,62281 |
| 54 | 42 | 1 | 10,77 | 10,8 | 0,277778 |
| 55 | 57 | 1 | 9,87 | 10,3 | 4,174757 |
| 56 | 62 | 1 | 9,79 | 9,8 | 0,102041 |
| 57 | 61 | 1 | 6,59 | 9,8 | 32,7551 |
| 58 | 60 | 1 | 9,06 | 9,8 | 7,55102 |
| 59 | 47 | 1 | 10,78 | 10,8 | 0,185185 |
| 60 | 32 | 1 | 11,35 | 11,4 | 0,438596 |
| 61 | 48 | 1 | 9,22 | 10,8 | 14,62963 |
| 62 | 59 | 1 | 4,389 | 10,3 | 57,38835 |

ALD: axonal loss degree; SFP- FMG: small fiber pathology in patients with fibromyalgia syndrome; IENFD: intraepidermal nerve fiber density; NV: normal value cutoff according to age and sex stratification.
