# Supplementary material for: Comparative Analysis of Small Nerve Fiber Density in Fibromyalgia Syndrome and Small Fiber Neuropathy
Source: Biomedicines. 2025 Aug 29;13(9):2109. doi: 10.3390/biomedicines13092109 (PMC12467328; doi:10.3390/biomedicines13092109)
Supplement: Supplementary file 1 [file biomedicines-13-02109-s001.zip › Supplementary Table S2.pdf]

Supplementary Table S2. Distal ALD calculation in patients with SFN.

	age	Sex (0=M, 1=F)	IENFD	NV cutoff	ALD
1	39	0	9,44	10,3	8,349515
2	70	0	7,85	8,2	4,268293
3	67	0	5,89	8,7	32,29885
4	68	1	3,87	9,8	60,5102
5	64	1	7,48	9,8	23,67347
6	52	0	6,19	9,3	33,44086
7	76	1	7,07	9,2	23,15217
8	59	1	1,94	10,3	81,16505
9	56	0	5,91	9,3	36,45161
10	62	1	7,6	9,8	22,44898
11	35	1	6,59	11,4	42,19298
12	72	0	4,579	8,2	44,15854
13	57	0	5	9,3	46,23656
14	65	1	9,47	9,8	3,367347
15	37	0	9,49	10,3	7,864078
16	64	1	4,24	9,8	56,73469
17	66	0	6,83	8,7	21,49425
18	67	0	4,72	8,7	45,74713
19	51	1	5,25	10,3	49,02913
20	32	1	6,27	11,4	45
21	34	1	11,07	11,4	2,894737
22	71	1	4,611	9,2	49,88043
23	24	1	10,59	11,9	11,0084
24	67	0	3,75	8,7	56,89655
25	54	1	8,29	10,3	19,51456
26	36	1	9,28	11,4	18,59649
27	48	0	4,78	9,8	51,22449
28	53	0	7,34	9,3	21,07527
29	81	0	5,83	8,2	28,90244
30	52	0	7,89	9,3	15,16129
31	39	0	7,06	10,3	31,45631
32	79	0	4,58	8,2	44,14634
33	46	0	8,86	9,8	9,591837
34	54	0	8,04	9,3	13,54839
35	58	0	3,81	9,3	59,03226
36	45	1	10,6	10,8	1,851852
37	59	1	7,12	10,3	30,87379
38	39	1	10,96	11,4	3,859649
39	76	1	2,68	9,2	70,86957
40	61	1	8,1	9,8	17,34694
41	45	1	8,89	10,8	17,68519
42	47	0	4,6	9,8	53,06122
43	67	0	5,78	8,7	33,56322
44	58	0	4,83	9,3	48,06452
45	64	0	4,89	8,7	43,7931
46	62	0	7,48	8,7	14,02299
47	42	1	4,38	10,8	59,44444
48	66	0	2,1	8,7	75,86207
49	51	0	8,34	9,3	10,32258
50	60	0	3,95	8,7	54,5977
51	42	1	1,39	10,8	87,12963
52	51	1	7,96	10,3	22,71845
53	66	0	4,18	8,7	51,95402
54	60	1	7,17	9,8	26,83673
55	53	0	8,37	9,3	10
56	52	1	6,74	10,3	34,56311
57	56	1	6,93	10,3	32,71845
58	34	1	6,76	11,4	40,70175
59	54	0	4,867	9,3	47,66667
60	39	0	7,39	10,3	28,25243
61	47	1	9,18	10,8	15

62	49	1	8,63	10,8	20,09259
63	65	1	8,03	9,8	18,06122
64	58	0	8,84	9,3	4,946237
65	80	1	5,03	9,2	45,32609
66	71	1	7,57	9,2	17,71739
67	57	1	5,27	10,3	48,83495
68	58	1	9	10,3	12,62136
69	42	1	8,23	10,8	23,7963
70	50	1	5,17	10,3	49,80583
71	49	1	7,12	10,8	34,07407
72	77	0	2,57	8,2	68,65854
73	50	1	4,84	10,3	53,00971
74	55	1	6,535	10,3	36,5534
75	45	0	5,41	9,8	44,79592
76	43	0	9,44	9,8	3,673469
77	19	1	9,74	11,9	18,15126
78	54	1	5,46	10,3	46,99029
79	55	1	5,6	10,3	45,63107
80	50	0	7,45	9,3	19,89247
81	68	1	6,42	9,8	34,4898
82	46	1	6,13	10,8	43,24074
83	20	1	9,4	11,9	21,0084
84	59	1	6,31	10,3	38,73786
85	35	1	8,46	11,4	25,78947
86	55	1	5,63	10,3	45,33981
87	49	0	1,87	9,8	80,91837
88	62	1	8,27	9,8	15,61224
89	57	1	7,5	10,3	27,18447
90	24	1	11,07	11,9	6,97479
91	45	1	6,62	10,8	38,7037
92	59	0	4,93	9,3	46,98925
93	63	1	5,02	9,8	48,77551
94	73	0	5,01	8,2	38,90244
95	65	0	4,1	8,7	52,87356
96	66	1	9,38	9,8	4,285714
97	72	1	5,12	9,2	44,34783
98	55	1	4,95	10,3	51,94175
99	76	1	4,45	9,2	51,63043
100	74	1	6,79	9,2	26,19565
101	43	0	6,83	9,8	30,30612
102	60	1	1,33	9,8	86,42857
102	69	0	6,37	8,7	26,78161
104	55	0	7,01	9,3	24,62366
105	65	1	0,1	9,8	98,97959
106	56	0	4,35	9,3	53,22581
107	67	1	3,78	9,8	61,42857
108	64	0	8,35	8,7	4,022989
109	61	0	4,5	8,7	48,27586
110	49	0	4,38	9,8	55,30612
111	72	0	6,7	8,2	18,29268
112	53	1	4,39	10,3	57,37864
113	78	1	6,77	9,2	26,41304
114	47	0	2	9,8	79,59184
115	53	0	6,35	9,3	31,72043
116	52	0	6,4	9,3	31,1828
117	51	1	6,79	10,3	34,07767
118	69	1	5,15	9,8	47,44898
119	39	1	6,9	11,4	39,47368
120	50	0	6,34	9,3	31,82796
121	56	1	9,28	10,3	9,902913
122	56	1	9,896	10,3	3,92233
123	64	1	5,96	9,8	39,18367
124	44	0	5,76	9,8	41,22449
125	55	0	9,1	9,3	2,150538
126	66	1	0,41	9,8	95,81633

127	65	0	5,05	8,7	41,95402
128	64	1	5,5	9,8	43,87755
129	56	1	9,15	10,3	11,16505
130	62	0	0,83	8,7	90,45977
131	63	0	7,74	8,7	11,03448
132	69	0	1,08	8,7	87,58621
133	39	1	4,22	11,4	62,98246
134	43	0	9,5	9,8	3,061224

ALD: axonal loss degree; SFN: small fiber neuropathy; IENFD: intraepidermal nerve fiber density; NV: normal value cutoff according to age and sex stratification
