# Supplementary material for: Comparative Analysis of Small Nerve Fiber Density in Fibromyalgia Syndrome and Small Fiber Neuropathy
Source: Biomedicines. 2025 Aug 29;13(9):2109. doi: 10.3390/biomedicines13092109 (PMC12467328; doi:10.3390/biomedicines13092109)
Supplement: Supplementary file 1 [file biomedicines-13-02109-s001.zip › Supplementary Table S3.pdf]

Supplementary Table S3. Subgroup analysis of patients with SFN sorted by etiology.

	Distal ALD	*p*
Diabetes (n=25)	47.37 (21.41)	**<0.0001**
Autoimmune (n=30)	36.69 (21.12)	**<0.0001**
Infectious (n=12)	23.79 (15.3)	0.0573
Genetic (n=38)	37.54 (22.33)	**<0.0001**
Idiopathic (n=29)	29.1 (22.96)	**0.0018**

Each value is expressed as mean (SD).

SFN: small fiber neuropathy; ALD: axonal loss degree; p: comparison with the SFP-FMG group, using the Mann–Whitney test.
