# Supplementary figures and images for: Comparative Analysis of Small Nerve Fiber Density in Fibromyalgia Syndrome and Small Fiber Neuropathy
Source: Biomedicines. 2025 Aug 29;13(9):2109. doi: 10.3390/biomedicines13092109 (PMC12467328; doi:10.3390/biomedicines13092109)

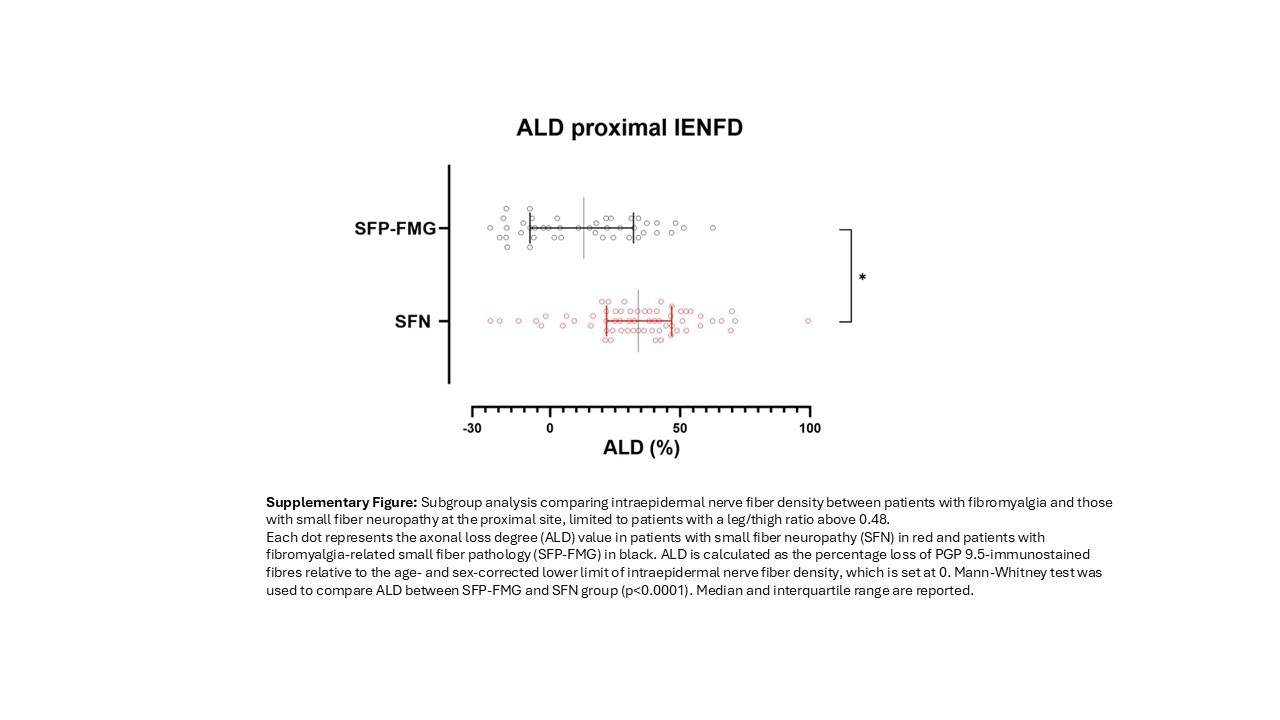

Supplement: Supplementary file 1 [file biomedicines-13-02109-s001.zip › Supplementary Figure S1.jpg]
